# Electrical contacts to individual SWCNTs: A review

**DOI:** 10.3762/bjnano.5.229

**Published:** 2014-11-21

**Authors:** Wei Liu, Christofer Hierold, Miroslav Haluska

**Affiliations:** 1 Micro and Nanosystems, Department of Mechanical and Process Engineering, ETH Zurich, CH-8092 Zurich, Switzerland

**Keywords:** charge carrier transport, CNFET, electrical contact, metal–SWCNT interface, SWCNT

## Abstract

Owing to their superior electrical characteristics, nanometer dimensions and definable lengths, single-walled carbon nanotubes (SWCNTs) are considered as one of the most promising materials for various types of nanodevices. Additionally, they can be used as either passive or active elements. To be integrated into circuitry or devices, they are typically connected with metal leads to provide electrical contacts. The properties and quality of these electrical contacts are important for the function and performance of SWCNT-based devices. Since carbon nanotubes are quasi-one-dimensional structures, contacts to them are different from those for bulk semiconductors. Additionally, some techniques used in Si-based technology are not compatible with SWCNT-based device fabrication, such as the contact area cleaning technique. In this review, an overview of the investigations of metal–SWCNT contacts is presented, including the principle of charge carrier injection through the metal–SWCNT contacts and experimental achievements. The methods for characterizing the electrical contacts are discussed as well. The parameters which influence the contact properties are summarized, mainly focusing on the contact geometry, metal type and the cleanliness of the SWCNT surface affected by the fabrication processes. Moreover, the challenges for widespread application of CNFETs are additionally discussed.

## Review

### Introduction

The unique crystalline and electronic structure of single-walled carbon nanotubes (SWCNTs) afford extraordinary mechanical properties [1–3] as well as thermal and electrical conductivity [4–5], enabling ballistic charge carrier transport up to the microscale at room temperature [6–7]. The emergence of carbon nanotube field-effect transistors (CNFETs) using SWCNTs as the device channel provides a possible solution to the problem of undesirable, short-channel effects occurring in metal oxide semiconductor field-effect transistors (MOSFETs) on the order of tens of nanometers [8]. Furthermore, the diameter-normalized charge carrier density in sub-10 nm CNFETs is four times higher than for silicon-based devices [9]. Recently, the concept of a CNFET-based single processor was successfully implemented by Shulaker et al. [10]. These achievements reflect the great progress in fabrication technology that is advancing carbon nanotube technology closer to reality.

For SWCNT-based devices, the nanotubes must be connected to electrical circuitry, which is typically achieved by contacting the SWCNTs with metal. Due to possible low channel resistance (or even ballistic conduction), the metal–nanotube contact resistance can dominate the performance of these transistors. Devices with a low Schottky barrier height (SBH) or even with Ohmic contacts are often required. The principles of charge carrier transport developed for Si-based devices must be reconsidered for SWCNT-based devices due to the reduced dimensionality of the metal–SWCNT interface. In this review, the technological progress in the improvement of the electrical performance of metal–SWCNT contacts in CNFETs is reviewed. The approaches used to characterize the contact performance using two-, four- and multi-terminal measurements are described. The role of the contact geometry, metal type, cleanliness of the SWCNT surface, and post-metallization annealing process and their effect on the device with respect to resistance are summarized. Finally, the challenges in obtaining high-performance CNFETs are discussed, covering the reproducibility, long term stability of electrical contact properties, and large-scale fabrication options.

### Carrier transport mechanism at metal–SWCNT interface

When a metal and a semiconductor are brought into intimate contact, the electric charges are redistributed to reach equilibrium at the metal–semiconductor interface due to the difference in their material work functions. Because of the charge redistribution, a Schottky barrier is formed at the metal–semiconductor contact if the position of the Fermi level of the metal lies between the edges of the semiconductor conduction and valence bands. This barrier impacts charge carrier transport through the electrical contact. Ideally, for a preselected semiconductor, the height of the Schottky barrier should depend on the work function of the chosen metals. In fact this rule is commonly violated for bulk semiconductors due to the existence of metal induced gap states (MIGS) at the metal–semiconductor interface [11–12]. For bulk semiconductors, MIGS cause a dipole sheet at the metal–semiconductor interface and lead to the so-called Fermi level pinning effect, that is, when the Fermi level tends to be fixed at a constant position in the band gap of the semiconductor [13]. The net result is that the Schottky barrier height is less sensitive to the work function difference between the metal and the semiconductor than theoretically expected.

**Figure 1:**
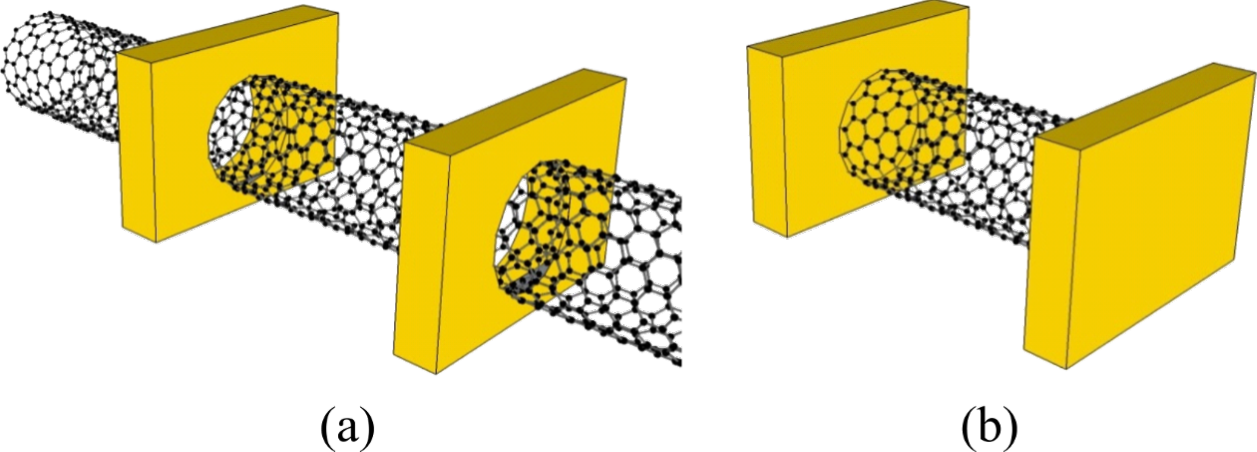
Contact geometries: (a) side-bonded and (b) end-bonded contact configurations.

**Figure 2:**
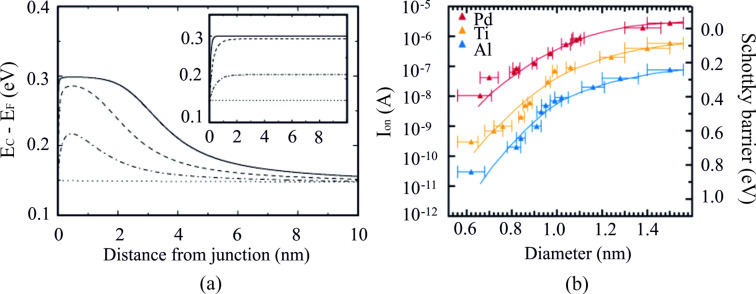
The role of the Fermi level pinning effect at the metal–semiconductor interface. a) Simulation of band bending at the metal–SWCNT (semiconducting SWCNT, diameter: 1.4 nm) interface for a low work function metal. The dotted, dash-dotted, dashed, and solid lines correspond to the different pinning strength 0, 0.01, 0.1, and 1 state/(atom eV), respectively. The pinning effect for the planar junction is shown in the inset for comparison using the same simulation parameters. b) The experimental results of the on current as a function of CNT diameter with different metals. The clear dependence of the Schottky barrier height on the type of metal and the SWCNT diameter is observed. This indicates that no Fermi level pinning effect exists at the metal–SWCNT interface. Figure reprinted with permission from (a) [14] copyright 2000 American Physical Society, (b) [15] copyright 2005 American Chemical Society.

However, the situation is different for the 1D, nanoscale, metal–semiconductor interfaces. The band bending structures for two types of contact geometries have been studied. For a SWCNT with an end-bonded contact (see the configuration shown in Figure 1b), MIGS cause a dipole ring instead of a dipole sheet at the metal–SWCNT interface, which only locally influences the band structures of SWCNTs within a few nanometers (Figure 2a) [14]. In contrast, for the planar contact, the dipole sheet affects the semiconductor band structure over a relatively longer distance (shown in the inset of Figure 2a).

For SWCNT with a side-bonded contact (configuration shown in Figure 1b), it was predicted as well that Fermi level pinning plays a minor role in the charge carrier injection effect at the electrical contact [16]. The experimental results [17] are consistent with this theoretical prediction [16]. Chen et al. [15] also reported that the Schottky barrier height decreased with increasing SWCNT diameter for the metals tested (Figure 2b). Meanwhile, for SWCNTs of the same diameter, a clear difference in electrical current (and Schottky barrier height) was observed for the contacts fabricated with Pd, Ti or Al [15] (Figure 2b).

As a result, for a given SWCNT, the height of the Schottky barrier can be adjusted by choosing the proper type of metal for the electrical contact. The work function of SWCNTs has been determined by the thermionic emission method to be 4.7–4.9 eV [18]. By choosing a metal with a high or low work function, it is possible to prepare the Schottky barrier favoring hole or electron conduction, respectively. Therefore, the types of transistors can be obtained by choosing the appropriate metal, ranging from fully p-type to ambipolar and to fully n-type.

Assuming the intrinsic Fermi level of SWCNTs is in the middle of the band gap (i.e., the intrinsic work function of the SWCNTs is approximated as the electron affinity plus half of the bandgap: 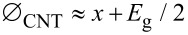), the corresponding Schottky barrier height for a metal with a low work function can be estimated as

[1]
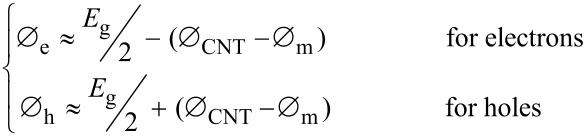


where 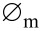 and 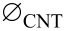 represent the work function of metal and SWCNT, respectively, and *E*_g_ is the band gap of the SWCNT, which is inversely proportional to its diameter (approximated as 0.71 ≈ 1.1/*d* [19–21]).

**Figure 3:**
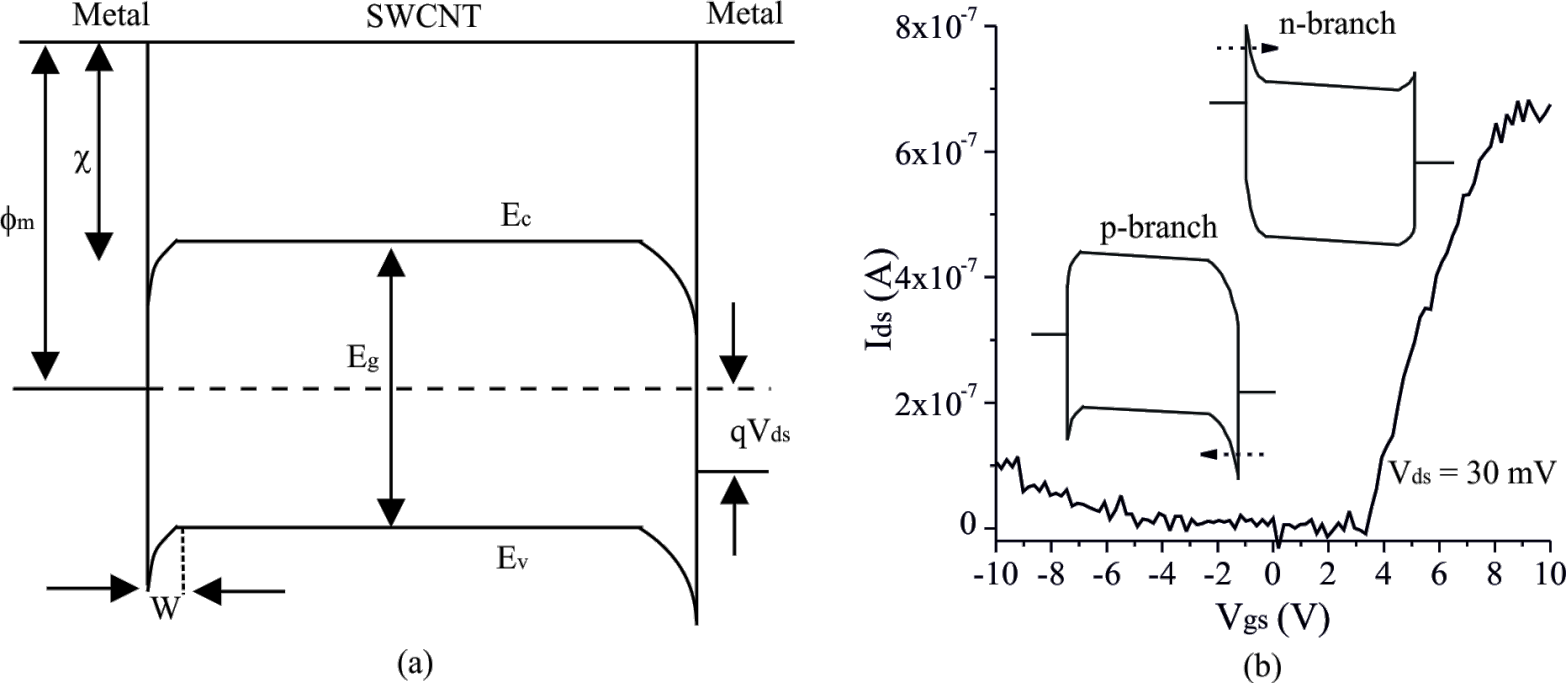
The energy band diagram of a CNFET. a) The band bending effect at the metal–SWCNT interface for a metal with a low work function. A positive voltage bias is applied between drain–source electrodes and the gate bias is set to zero. b) The transfer characteristics of an n-type CNFET. Inset sketches show the Schottky barrier width modulation with respect to the different bias conditions from the gate electrodes.

For a CNFET with a Schottky barrier (SB) at the contacts, the electrical current through the barrier contains the contributions from two components: the thermionic emission current (over the Schottky barrier) and the tunneling current (through the depletion region) [22]. The band bending diagram at the metal–SWCNT interface is illustrated in Figure 3a, assuming the drain–source electrode is biased with a positive voltage and the gate electrode is set to zero voltage potential. The electrical modulation of the CNFET conductance is the consequence of the Schottky barrier width (*W*) modulation [23]. The shape of Schottky barrier can be modulated by the gate and drain–source voltages, therefore, the transmission probability, *T*(*E*), of carriers across the SB is determined by the electric field induced by both the gate and drain–source bias. This effect is illustrated in the inset of Figure 3b, showing typical transfer characteristics of an n-type CNFET. Under a low gate bias (less than the threshold voltage), the Schottky barrier is too high and wide to allow the charge carrier flow over or to tunnel through the barrier. When the gate voltage is positively increased (greater than the threshold voltage), the bands bend downward and narrow the Schottky barrier width, enabling more electrons to tunnel through (the n-branch shown in the inset of Figure 3b). In this regime, the current grows exponentially with an increase in the gate bias. As the gate voltage increases further, the gate-field-induced band bending effect slows down causing the source–drain current to become “saturated” beyond a certain gate voltage. The bands bend upward when a negative voltage is applied to the gate electrode and the Schottky barrier for holes is reduced. Under this condition, the activated hole carriers tunnel through the Schottky barrier (the p-branch shown in the inset of Figure 3b). Due to this, a current (for holes) is observed under a negatively biased gate voltage.

### Characterization methods

The overall resistance (*R*_tot_) of a CNFET includes the contact resistances (2*R*_c_) and the device channel resistance (*r*_ch_*L*_ch_) given as [24]:

[2]
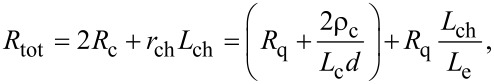


where the quantum resistance for a SWCNT is defined as *R*_q_ = *h*/4e^2^, *r*_ch_ is the channel resistance per unit length, *R*_c_ is the single contact resistance, ρ_c_ is the specific contact resistivity, *L*_c_ and *L*_ch_ represent the contact width and the channel length, respectively, *d* is the diameter of the SWCNT, and *L*_e_ is the mean free path for carrier scattering (which varies with the temperature [25]). *L*_e_ is in the range of few hundreds nanometers [24,26] to a few micrometers [27], which reflects the quality of the SWCNTs (defects or imperfections) influenced by the SWCNT synthesis and the device fabrication processes.

The overall resistance for the on-state of CNFETs obtained from the experiments are typically much higher than the theoretically calculated value for CNFETs with a 1D channel (*h*/4e^2^ = 6.5 kΩ) for SWCNTs [28]. Thus, it is necessary to determine the resistance contributions from the electrical contacts and the device channels. The common experimental techniques used to determine the contribution of the individual resistance components are: 1) extraction of the Schottky barrier height from the CNFET transfer characteristics measured at different temperatures, 2) estimation of the channel resistance through a four-terminal measurement, and 3) evaluation of the contact and channel resistances by a multi-terminal experiment using the transmission line model.

### Determination of the Schottky barrier height

As mentioned previously, CNFETs (using SWCNTs as the active channel) are SB-modulated devices (SB-CNFETs). The width of the Schottky barrier can be determined by locally excited photo-voltage experiments [29] or by checking the bright segment in the SEM image [30]. The width of the Schottky barrier is modulated by the applied electric field from the gate and source–drain electrodes [23], but the height of Schottky barrier is mainly determined by the work function difference between metal and carbon nanotube. To some extent, the height of the Schottky barrier reflects the intrinsic property of electrical contact. The approaches used to evaluate the height of the Schottky barrier are detailed in this section.

Although a capacitance–voltage (C–V) measurement was developed to extract the Schottky barrier height for silicon planar devices [31], it requires a more sophisticated setup for CNFETs due to the small capacitance in the metal–SWCNT contact area [32]. Current–voltage (I–V) measurements performed at different temperatures (also called the thermal activation energy method) are more suitable for extracting the Schottky barrier height at the metal–SWCNT contacts in CNFETs [33–34]. According to the thermionic emission theory, the thermionic current through the metal–semiconductor contact is proportional to the measurement temperature [35]. The SB height can be determined from the following equation [36]:

[3]
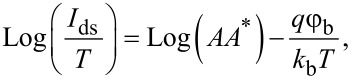


where *A* is the contact area, A* is the effective Richardson constant, *q* and *k*_b_ represent the elementary charge and Boltzmann constant, respectively, *q*φ_b_ is the activation energy, and *T* is the temperature.

**Figure 4:**
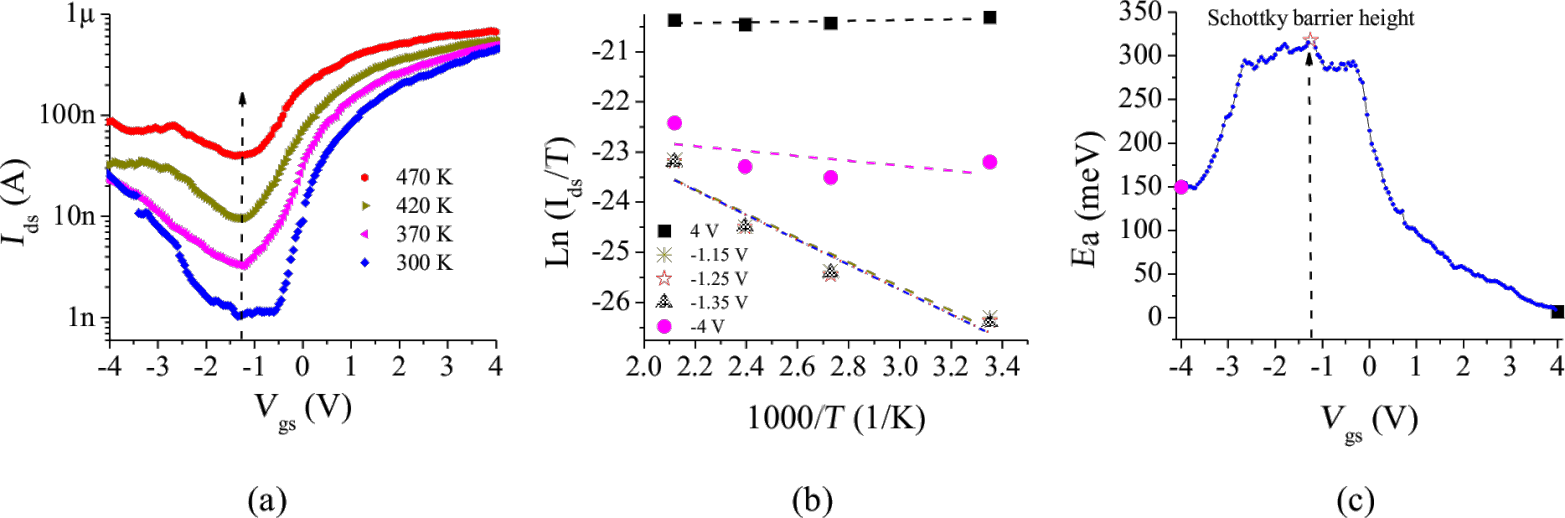
The procedures for determining the Schottky barrier height. (a) CNFET transfer characteristics measured at different temperatures. (b) Arrhenius plot with linear fits for different gate biases, *V*_gs_. For the better visualization, only the data points obtained for five different gate voltages: 4 V, −1.15 V, −1.25 V, −1.35 V, −4 V are shown. (c) Activation energy (*E*_a_) as a function of gate bias (*V*_gs_) estimated from (a). The highlighted data points represent the activation energies derived from the corresponding slopes of linear fits in (b).

Figure 4a shows the transfer characteristics of the same CNFET obtained at different temperatures. The off-state current reveals a stronger temperature dependence because it mainly represents the thermally activated carrier transport (where charge flow over the Schottky barrier dominates). The trend is more obvious in the Arrhenius plot (Figure 4b). The maximum slope is obtained at a certain gate bias where the minimum current is obtained. This corresponds to the flat band condition, that is, no band bending occurs at the metal–SWCNT interface at the source electrode for this condition [36]. On the other hand, the currents measured at the more positive and negative biases reveal only a weak temperature dependence due to carrier transport dominated by tunneling. Utilizing this characteristic, the activation energy (*q*φ_b_, Figure 4c) is extracted according to Equation 3. Its maximum value corresponds to the Schottky barrier height. The activation energy becomes smaller when the energy bands are no longer in the flat band condition because of the added contribution of the tunneling current through the thinner part of the Schottky barrier.

It is worth noting that the Schottky barrier height could be influenced by the surrounding atmosphere. As reported in [37–38], the oxygen molecules at the metal–CNT contact had an effect on the Schottky barrier height. Therefore, the measurement conditions (in vacuum or in air) should be considered during the electrical characterization.

### Determination of the contact and channel resistances

**Figure 5:**
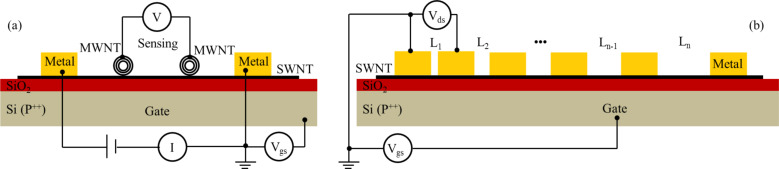
Schematics of different approaches for characterizing the electrical contacts of CNFETs. a) An optimized configuration for the four-terminal measurement by using MWCNTs as the voltage sensing electrode according to the reference [26]. b) Multiple contacts are defined on the same individual SWCNT with different channel lengths. Potential drops can be measured between any two electrodes.

**Four-terminal measurement:** The four-terminal measurement is often used to determine the sheet resistance of thin films. The contact resistance can be extracted indirectly by subtracting the thin film resistance from the overall resistance. Two pairs of terminals are contacted to the target material and a constant current (*I*_con_) is forced from two outside terminals and the voltage drop (*V*_sen_) along the object is measured between the two inner “sensing” terminals. This method has been adapted to determine the resistance in CNFETs [39–42]. The channel resistance between the two inner terminals is determined by *R*_ch_* = V*_sen_/*I*_con_. The contact resistance can be obtained by subtracting the channel resistance from the overall resistance measured from the inner electrode pair. However, those electrodes can cause deformation of the SWCNT structure and/or induce additional current scattering under the contacts [43–44], which can perturb the carrier transport along the SWCNT. For the bottom contact configuration, the nanotube deformation was also detected near the edges of the electrodes where the SWCNTs passed from the top electrodes to the substrate [45]. Therefore, it is necessary to set up a suitable measurement platform to eliminate the negative influence of the contacts on the nanotubes. Gao et al. [26] presented an approach to solve this problem by using noninvasive electrodes. As shown in Figure 5a, two MWCNTs acting as the sensing electrodes were placed on a SWCNT by an AFM manipulator. As a result, the additional barriers caused by the deformation of the SWCNT were avoided [26].

**Transmission line model:** CNFETs with longer channel lengths exhibit a higher on-resistance due to the electron scattering effect along the channels [46]. This could be caused by intrinsic structural defects or by the mechanical bending of CNTs lying on the substrate as well as by the chemical inhomogeneity induced during the fabrication process. The transmission line model (TLM) developed for planar devices [47] provides an approach to determine both the contact resistance and the channel resistance. The applicability of this approach was extended to nanoscale devices, such as CNFETs [24,48]. As shown in Figure 5b, multi-electrodes are defined on the same SWCNT. The overall resistance is measured for CNFETs with different channel lengths. For the CNFET with a short channel length made from a high quality SWCNT and measured at a low source–drain bias, the resistance contributed from the device channel plays a minor role in the total resistance when approaching ballistic transport. The total resistance (*R*_tot_) is determined as shown in Equation 2. Based on the assumption that each contact is of equal quality and the nanotube is consistent along the entire length, the contact resistance and the channel resistance can be determined by plotting the overall resistance versus the channel length. The corresponding resistances can be determined from the intercept (2*R*_c_) and the slope (*r*_ch_) of the curve.

### Factors influencing the contact properties

The device architecture and characterization conditions often vary from device to device, as prepared by different research groups. For this reason, we will discuss the results obtained by different groups separately to better understand the effect of contact geometry, metal type, and the nanotube cleanliness, as well as the post-metallization annealing treatment.

#### Contact geometry

The contact geometries of CNFETs can be classified into two types: end-bonded and side-bonded contacts (as shown in Figure 1a,b). For end-bonded contacts, metal leads are directly touching the open ends of nanotube. In this case, the defect sites (dangling bonds) on the ends of the CNTs are intended to provide additional reaction sites to greatly increase the interaction energy between the metal and the CNT [49]. As an example, nanotube/carbide heterojunctions (such as TiC) can be formed at an optimized temperature [50]. This has been shown to be a great advantage for MWCNT interconnects. As demonstrated in [51], the caps of vertically (with respect to the horizontal substrate) aligned MWCNTs were removed by chemical mechanical polishing (CMP) followed by metal deposition to form the top electrodes. By this approach, the inner shells of MWCNTs are also contacted by metal, thereby enhancing the carrier transport capability. However, this fabrication process cannot be easily applied to horizontally aligned SWCNTs. Instead, side-bonded contacts are implemented and widely used in SWCNT-based device fabrication. SWCNTs can be deposited on the predefined metal electrodes, or the other way around. The effective contact area between the metal and SWCNT allows for the charge carrier transport capability. As a result, a term called the transfer length, *L*_T_, is introduced to quantify the carrier transfer distance under the electrical contact. Nosho et al. [48] experimentally determined the transfer length to be a few tens of nanometers. Investigations by another group [24] showed that the contact resistance increased exponentially when the contact length decreased from 200 to 20 nm. This inconsistency could be caused by the different process conditions, such as the contact materials (Au vs Pd), annealing treatment, and the different properties of the individual SWCNTs used. The results imply that the carrier transport mainly occurs at or close to the edge of the metal–SWCNT contact.

**Figure 6:**
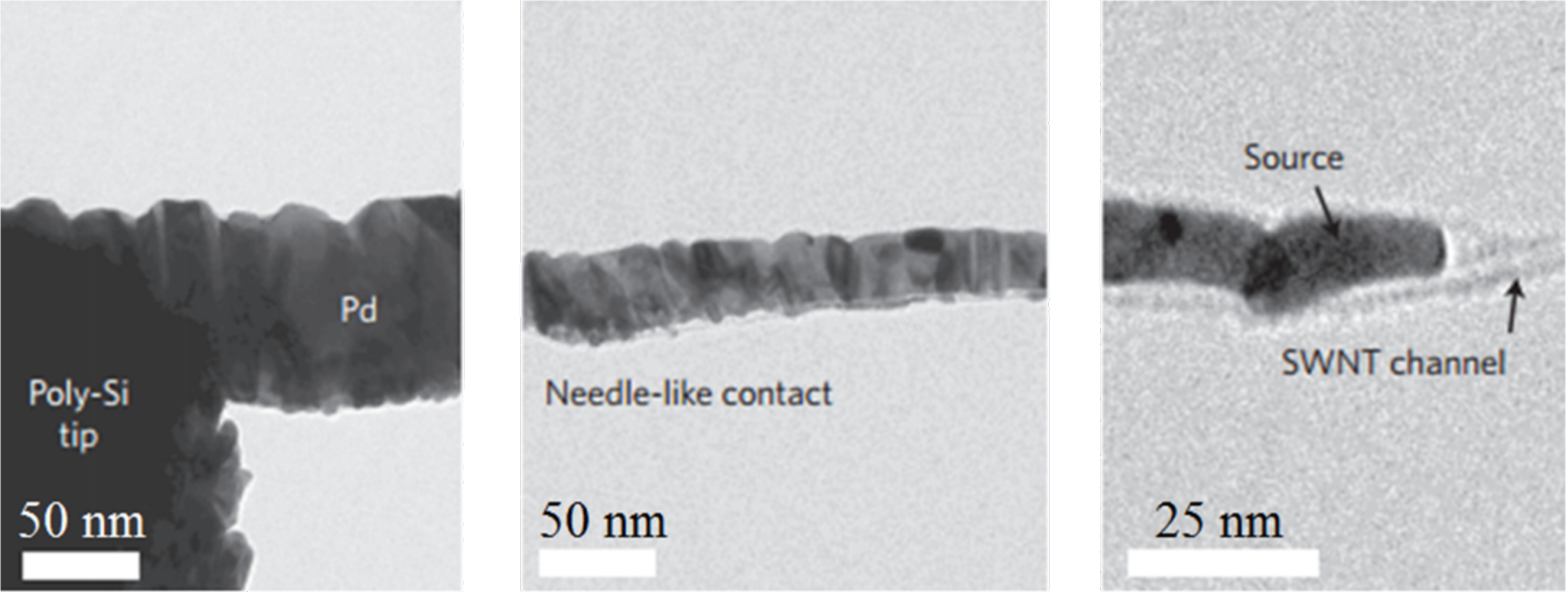
Needle-like side-bonded contact. Reprinted with permission from [53]. Copyright 2010 Macmillan Publisher.

Another crucial factor which could influence the charge carrier transport at the metal contact is the shape of the electrode. It was shown [52] that a needle-like contact is preferred to improve the gate-field efficiency and thus enhance the charge carrier transport at the metal–SWCNT interface. This approach has been experimentally implemented by Muoth and coworkers [53]. As shown in Figure 6, the thickness of the metal coating on the SWCNT gradually decreases from tens of nanometers to a few nanometers. Owing to the nano-tapered contacts, a remarkable subthreshold swing (230 mV dec^−1^ using air as the gate dielectric) was obtained for a device fabricated on a suspended SWCNT using a 3 µm air gap as the dielectric material between the gate electrode and the transistor channel.

#### Contact metal

**Work function and wettability:** The presence of a Schottky barrier at the metal–SWCNT contact largely impacts the CNFET performance. Since the pinning effect at the metal–CNT interface is nearly negligible [14,16], the height of the Schottky barrier could be minimized by choosing an appropriate metal with a relatively high (low) work function for high performance p-type (n-type) CNFETs. The Schottky barrier height is also inversely proportional to the diameter of the SWCNT. The discussion in this section will be focused on how the device performance is affected by the metal properties, for a given SWCNT.

High-performance n-type CNFETs were achieved by using Sc contacts [54], where the device conductance reached 0.5∙*G*_0_ (1/*R*_q_ = *G*_0_ = 154 µS) measured at room temperature. However, the strong propensity of oxidation for low work function metals cause low yield and degradation in the reproducibility of device performance. Recently, Shahrjerdi et al. [55] reported a remarkable improvement in n-type transistors by using erbium (Er) as the contact metal. A high device yield was obtained through controlling the metal deposition conditions, such as the base pressure and deposition rate. For p-type CNFETs, Pd has proven to be the most appropriate metal, forming nearly Ohmic contacts with superb device yield [56].

**Figure 7:**
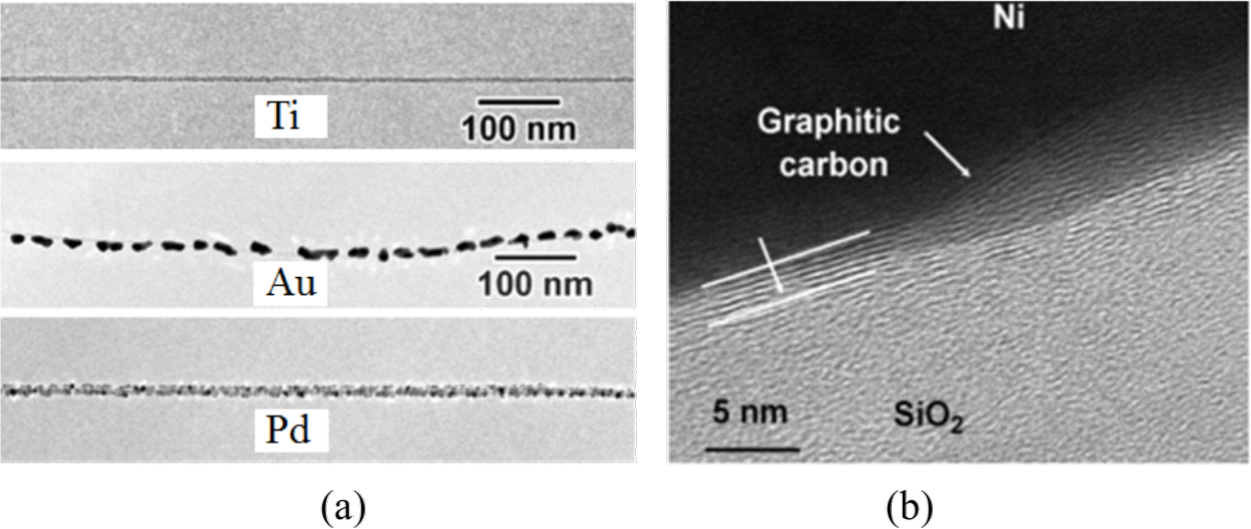
TEM images for (a) metal deposition on suspended, as-grown SWCNTs. Ti displays the best wettability to the SWCNT. Compared to Au, Pd showed better wettability to the SWCNT. (b) The graphitic carbon interfacial layer formed under a Ni layer through annealing treatment. Figure reprinted with permission from (a) [57] copyright 2000 American Institute of Physics and (b) [64] copyright IEEE Electron Devices Society.

Other than the work function differences between the metals and the SWCNTs, the wettability of metals to SWCNTs is another critical factor affecting the contact properties. Although Au and Pd have comparable work functions (shown in Table 1), Au–CNT contacts possess a higher on-resistance in most cases. This can be explained by the relatively poor wettability of gold to SWCNTs. As shown in Figure 7a, discrete Au nanoparticles are formed on suspended SWCNTs [57]. In contrast, Pd forms a nearly continuous coating on the SWCNT [57], which indicates a good adhesion to the sidewall of carbon nanotube. Another promising metal with a high work function is Pt. Although it possesses a work function higher than Pd, non-Ohmic contacts were observed from the CNFETs with Pt–SWCNT contacts [27]. The effective contact area of Pt on SWCNT is limited by the poor wettability of Pt [58]. All these experimental observations point to the importance of metal wettability to SWCNTs [56,58–59]. A metal with better wettability provides a greater contact interface area per unit length of the nanotube, which enhances the current injection capability. On the other hand, metals with poor wettability cause a discontinuous coating resulting in the presence of vacuum voids at the metal–CNT interface. Therefore, it is not surprising that Pd exhibits the best quality of electrical contacts owing to both properties, that is, a high work function as well as good wettability to the SWCNT [60]. As reported by Franklin et al. [24], the total resistance (including the channel and contact resistances) reached 6.6 kΩ for metallic SWCNT contacted by Pd. Ti also shows excellent wettability to SWCNTs (Figure 7a). Using Ti as a contact material may result in Ohmic contact [7] as well as Schottky contact [61]. The variation in results is probably caused by the easy oxidation of Ti [62]. As was presented in [27], a maximum of a 20% success rate for the formation of Ohmic contacts was obtained for Ti–SWCNT contacts. Obviously, the chemical inertness of the contact metal in air is another important requirement for forming reliable electrical contacts.

**Table 1:** Work function of various metals [63] (units: eV).

Metal	Work function	Metal	Work function	Metal	Work function	Metal	Work function

Er	3.0	Al	4.28	W	4.55	Au	5.1
Sc	3.5	Ti	4.33	Mo	4.6	Pd	5.12
Mn	4.1	Cr	4.5	Cu	4.65	Ni	5.15
Ag	4.26	Fe	4.5	Rh	4.98	Pt	5.65

**Carbon interfacial layer:** The imperfect wettability of the metal to the nanotube leads to the occurrence of atomic-level physical gaps between the metal and the nanotube, thus causing the presence of an additional barrier which hinders charge carrier injection at the contacts. Although Pt provides poor coverage of the nanotubes [59], the electrical contact could be improved by introducing a graphitic interfacial layer between the SWCNT and the Pt electrode. As pointed out in [65], Pt acts as a catalyst for hydrocarbon residues (inevitably present in the vacuum chamber) which can be graphitized by an annealing treatment above 607 °C. However, the uncertainty of hydrocarbon residue concentration in the process chamber constrains the reproducibility of fabrication of low-resistance contacts. Chai et al. [64] developed another approach to obtain a graphitic interfacial layer in a more controllable manner. Amorphous carbon was deposited between Ni (as catalysts for graphitization) and semiconducting SWCNTs. The amorphous carbon transforms to graphitic carbon by annealing at 850 °C (as shown in Figure 7b). By this approach, the effective contact area between the metal and the SWCNTs is increased, thus improving the charge carrier transport. In this way, the on-current was improved by one order of magnitude [64].

#### Cleanliness of SWCNT surface

**Figure 8:**
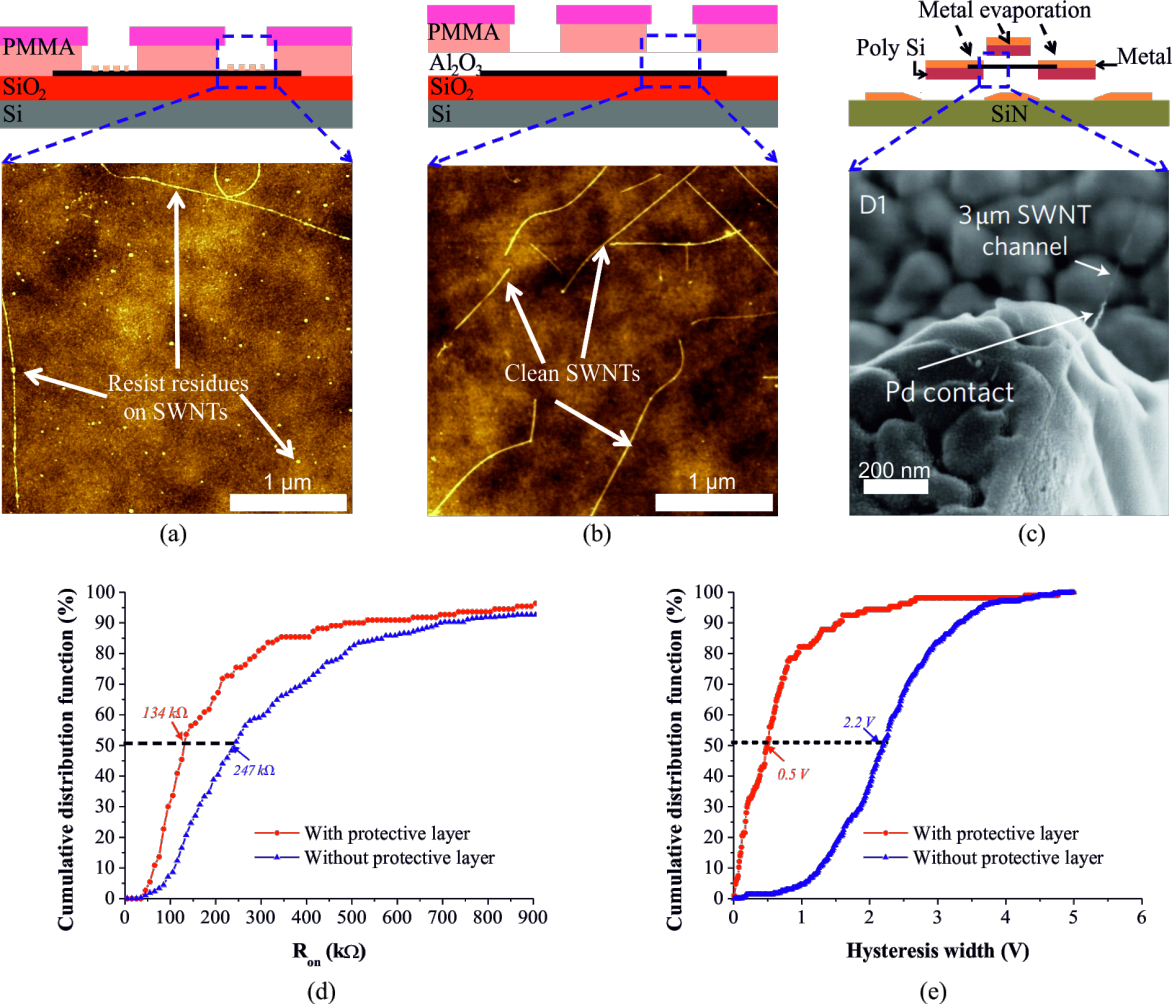
Inspection of the cleanliness of SWCNTs. a) AFM image obtained after developing a PMMA resist from the SiO_2_-supported SWCNTs. b) AFM image obtained after PMMA and following alumina removal from the contact area. c) SEM image of a Pd contact on a suspended SWCNT using a shadow mask process. d) The cumulative distribution function of on-resistance for n-type CNFETs. Red circles and blue triangles represent the experimental results with and without using the protective layer, respectively. e) The distribution of the hysteresis width for n-type CNFETs. Figure reprinted with permission from (c) [53] copyright 2010 Macmillan Publisher and (d,e) [71] copyright 2014 Elsevier.

It is challenging to avoid resist residue from resist-based lithographic fabrication processes on SWCNTs. Although oxygen plasma etching is an efficient approach to remove the resist residue in silicon-based device fabrication processes [66], this harsh treatment destroys the CNT structure [67]. Several different approaches have been explored to preserve the clean surface of SWCNTs. Optimizing the resist wet-stripping procedure only partly helped to improve removability of the resist residue from the contact area [68]. To better clean SWCNTs, a dry-cleaning technique was developed. As presented in [69], the resist residue on SWCNTs can be removed by annealing (350 °C) the sample in forming gas (95% N_2_, 5% H_2_). However, neither wet-cleaning nor dry-cleaning techniques are applicable to remove the resist residue from the contact area, as they would destroy the preferably undercut profile of the resists required for the subsequent metal lift-off process. Khamis et al. [70] introduced a buffer layer between SWCNTs and a novolac-based photoresist to prevent SWCNTs from directly contacting the photoresist. However, the relatively high on-resistance (250 ± 100 kΩ) indicates the necessity for further optimization. Recently, the authors [71] have investigated an approach by using a thin layer of alumina to protect the SWCNTs during the device fabrication. The results presented in Figure 8a,b show that the resist residue is dramatically reduced at the contact area by using an alumina protective layer. Owing to the improvement in cleanliness of the SWCNT surface, the median value of the on-resistance was reduced from 247 kΩ for unprotected SWCNTs (381 n-type CNFETs) to 134 kΩ for protected SWCNTs (110 n-type CNFETs) (Figure 8d) [71]. The median value of the hysteresis width also narrowed from 2.2 V to 0.5 V (Figure 8e) [71]. In addition, the widths (see the insets in Figure 8d,e) of the dispersions of on-resistance and the hysteresis width were reduced to 152 kΩ and 0.6 V, respectively. Another option to avoid the impact of contamination induced by the photo/e-beam resists is stencil lithography. As reported by Muoth et al. [53], hysteresis-free devices were achieved by using a shadow mask to define the electrical contacts (resist-free). In this process, SWCNTs were grown between two suspended poly-Si tips. Then an on-chip shadow mask covered the SWCNT to protect the device channel. With the assist of a shadow mask, the electrical contacts were formed by directly depositing metals onto the poly-Si tips, which were bridged by a nanotube (as shown in Figure 8c). This approach eliminates contamination from the photo/e-beam resists completely. However, the large-scale applicability is constrained by the complexity of the fabrication process. Nevertheless, other groups have developed wafer-scale stencil lithography processes which could be used as a scale-up process for patterning metal–nanotube contacts. For instance, Vazquez-Mena et al. [72] developed a stencil lithography process to fabricate metallic nanowires at the wafer level. Nanoslits with a width down to 70 nm were defined on a wafer level membrane made of low-stress silicon nitride. Nanowires were thus formed by depositing metal through the nanoslits without using any photo/e-beam resist.

#### Post-metallization annealing

The metal–SWCNT contact performance can be further improved by post-metallization annealing. With this approach, the following benefits can be attained: 1) the removal of possible organic contaminations from the metal–SWCNT contact area in the case of a bottom contact configuration, 2) the change in metal morphology at the contact area, and 3) the formation of covalent bonds between the nanotube and the metal, leading to the formation of metal carbides. Several combinations of metals and annealing conditions (temperatures, duration) have been investigated, as summarized in Table 2.

**Table 2:** Different annealing conditions for commonly used contact metals in CNFETs.

Contact metals (thickness in nm)	Types of nanotubes^a,b^	Contact geometry	Annealing conditions	Reduction of resistance

Cr/Au [70]	s-SWCNTs	top contact	300 °C in air for 1 h	≈2–3 orders of magnitude
Cr/Au = 5/50 [73]	s-SWCNTs	top contact	600 °C in Ar atmosphere for 10 min	reduced the SB height
Ti/Pd = 0.8/50 [74]	m-SWCNTs	top contact	≈177 °C in vacuum	≈1 order of magnitude
Ti = 50 [74]	m-SWCNTs	top contact	327 to 627 °C in vacuum	≈1–2 orders of magnitude
Ti probe [75]	CNT fiber	bottom contact	400 to 700 °C in vacuum	≈4 orders of magnitude
Ti = 50 [76]	s-SWCNTs	top contact	700 to 800 °C in Ar atmosphere for 30 s	≈2–3 orders of magnitude
Ti [50]	SWCNT matrix	bottom contact	≈970 °C in vacuum for 20 min	SB-contacts turned to Ohmic contacts
Ti/Pt = 0.8/30 [65]	m-SWCNTs	top contact	627 °C in vacuum	14 times (average)

^a^s-SWCNTs: semiconducting SWCNTs, ^b^m-SWCNTs: metallic SWCNTs.

**Figure 9:**
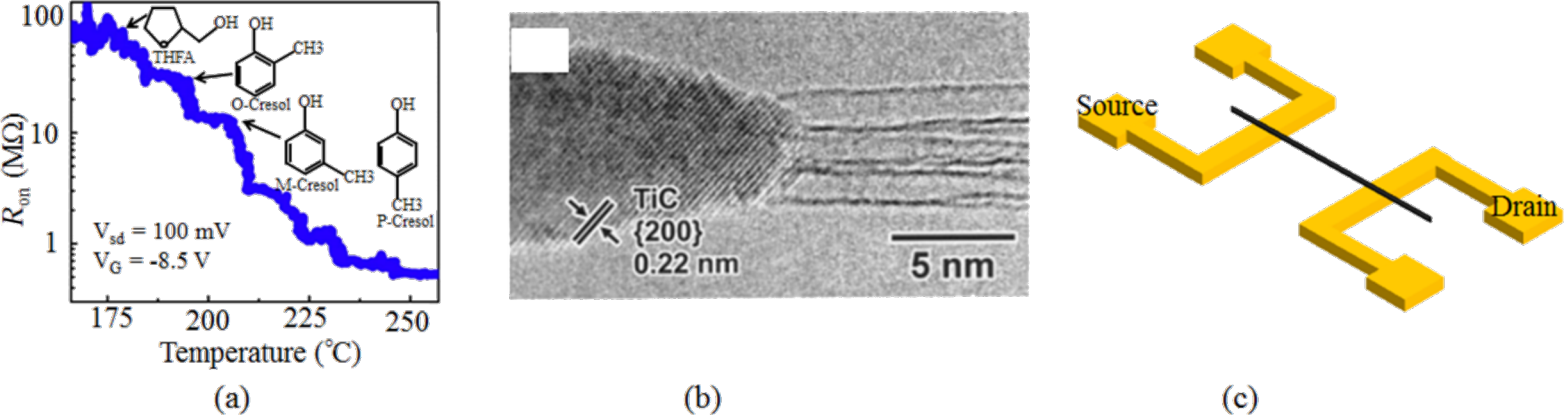
Improving the contact performance by post-metallization annealing. a) The process of removing contamination from the sidewalls of SWCNTs by annealing at modest temperatures. b) The TEM image shows the TiC formation through annealing Ti–SWCNT contacts above 800 ºC. c) The schematic of contact design using the Joule heating method for local annealing of the metal–SWCNT contact (using Pd as the contact metal) by pulsed current. Figure reprinted with permission from (a) [70] copyright 2011 Author(s) and (b) [50] copyright 1999 The American Association for the Advancement of Science.

Khamis et al. [70] pointed out that the device properties (for Au/Cr–SWCNT top contact) were improved at 300 °C, which helped to remove contamination attached to the channels of devices (Figure 9a). Another group [73] also highlighted the importance of the annealing treatment for Au/Cr–SWCNT contacts (top contact). The on-conductance was increased by 2–3 orders of magnitude through annealing at 600 °C in Ar atmosphere [73]. It was intended that the Au should penetrate the Cr layer and create a direct contact with SWCNTs after the annealing treatment [73]. Actually, evidence can be found in [77] which illustrates the inter-diffusion between the Cr (6 nm) layer and the Au (200 nm) as observed through the scanning Auger depth profile after 8 hours of annealing at 400 °C.

For Pd top contacts deposited on SWCNT, annealing at a relatively low temperature is preferred. As it was reported in [74], the overall resistance was reduced by three orders of magnitude after annealing at ≈177 °C. A further annealing step at 325 °C degraded the contact performance. This can be explained by the change of the Pd morphology, specifically by an increasing grain size of Pd at increased temperatures [78].

The performance of Ti–CNT top contacts [74] was investigated by annealing in vacuum in the range of 350–700 °C. The overall resistance was dramatically reduced by 1–2 orders of magnitude, owing to the chemical change at the metal–SWCNT–silicon oxide interface as well as the structure change of the Ti metal. Further annealing at a higher temperature has also been explored to form TiC by quenching the Ti contacts at 800–850 °C [76]. Thus, the conductance of ambipolar CNFET was improved by more than two orders of magnitude [76]. Zhang et al. [50] reported that the resistance of Ti-SWCNT bottom contacts was reduced by annealing the devices at temperatures greater than 800 °C due to the formation of nanorod-like TiC (Figure 9b). On the contrary, Tomimoto et al. [75] observed the gradually decreased conductance for Ti–SWCNT bottom contacts annealed at a temperature greater than 700 °C, which was explained as the reduction of the effective contact area between the conductor (Ti and TiC) and nanotube. The effective contact area could be the cross section or the partially carbidized side wall of SWCNT. However, it is difficult to experimentally inspect the effective contact area. Further investigation must be carried out.

The concept of electrically heating the contacts has also been explored. Figure 9c shows the schematic of using Joule heating to improve the contact properties. A constant or pulsed current [37,79–81] was applied through the electrodes. An instant heating locally in the contact area could remove the adsorbates remaining on the SWCNTs after wet deposition, thus reducing the tunneling barrier for carrier injection at the metal–SWCNT interface.

#### Challenges

Several milestones have been achieved regarding the improvement of electrical transport at the metal–CNT interface over the past decades. Nevertheless, there are still some challenges to be resolved before CNFET-based devices can be extensively used in a variety of applications. The most important challenges are reproducibility, predictability and long-term stability of device performance, as well as the feasibility of large-scale fabrication.

Comparing the performance of CNFETs obtained from the same fabrication batch, a large variation in the on-resistance was observed among thousands of devices [70]. The poor reproducibility of CNFET characteristics could be partially (but not entirely) explained by the variation in the CNT properties. Indeed, the current measured under the same conditions varies by nearly an order of magnitude for CNFETs fabricated using the same SWCNTs [82]. This suggests that the specific process steps during device fabrication, such as photo/e-beam resist contamination and the unclear effect caused by wet chemicals (developers, resist removers and etchants), affect the properties of SWCNTs. Therefore, the impact of the individual fabrication steps on SWCNT properties or the metal–SWCNT interface must be further investigated.

The long-term stability of CNFET transfer characteristics is another major concern for their applicability, which has been studied by several authors [83–85]. For devices with the Cr/Au (3 nm/20 nm) contacts, the electrical contacts were reported to be stable for 5 months without a passivation layer [83]. However, a contradictory observation was presented in [84], where severe degradation of electrical contact (Cr/Au: 2 nm/40 nm) was observed within a few days. The short lifetime of devices using Cr/Au (20 nm/75 nm) electrodes were reported in [85], where 92% of the devices did not function after 8 days. Even for the contact-passivated (by 1 µm PMMA) devices, 69% of them were not functional after a few days [85]. Without the passivation layer, the lifetime of devices using the same contact metals varies from a few months [41,83] to a few days [41,84]. Even for the passivated devices, the instability of CNFET performance was observed in [85–86]. The root cause of these seemingly contradictory results is unclear. It may be related to the different processes used for sample preparation or the inhomogeneity of SWCNT quality. This further highlights the necessity to clarify the factors influencing the stability of electrical contacts.

So far, the results presented in literature were mainly obtained from devices fabricated on the chip-scale. Results on wafer-scale fabrication are still lacking. Han et al. [87] have developed a process flow for wafer-scale CNFET fabrication, which is compatible with the current CMOS fabrication processes, but the SWCNTs require transfer to a target wafer. The physically and chemically induced impacts on the SWCNT properties which occur during the SWCNT transfer process must be further investigated. In particular, the organic contamination and gold etching issues must be examined. Park et al. [88] presented another wafer-level scalable process to disperse SWCNTs onto the pre-patterned substrate. A high density of SWCNTs were selectively deposited onto an array of HfO_2_ trenches by using an ion exchange surface chemistry approach. Tens of thousands of CNFETs were fabricated on the chip level with 78% yield. Chikkadi et al. [89] introduced a photolithography-based scalable fabrication process which provides a good platform for investigating the uniformity of CNFET performance on a large scale. To further optimize the capability of the fabrication process, more statistical analysis on wafer-level fabrication is needed.

## Conclusion

Both a theoretical understanding of the electrical transport in CNFETs and experimental techniques required for fabrication of high quality CNFETS with targeted characteristics have grown rapidly in recent years. Unlike for the case of planar contacts in Si-based devices, the Fermi level pinning effect at the metal–SWCNT interface is negligible. The Schottky barrier height at the metal–SWCNT contact can therefore be determined by selecting a metal with a suitable work function. So far, Pd is considered as the best metal for p-type CNFETs, owing to its high work function, good wettability to SWCNTs, as well as its stability in ambient conditions. Low work function metals were found to be extremely sensitive to oxidation, which leads to a low device yield. High-performance n-type CNFETs can be obtained by using Er as the contact material. The device yield can be largely improved by controlling the metal deposition conditions.

In addition to the choice of metal type, the CNFET performance can be further improved by annealing at modest temperatures, which may result in an improvement of the metal morphology or the formation of metal carbides at the contact interface. In the case of a solution-based SWCNT deposition onto metal electrodes, the annealing of the sample can reduce the amount of liquid residuals at the metal–SWCNT interface.

Another factor determining the metal–nanotube contact performance is the cleanliness of the metal–SWCNT interface. Using a protective layer to protect SWCNTs during the device fabrication helps to improve the contact performance.

The fabrication processes must be further optimized to improve the reproducibility and predictability of CNFETs to further their applicability. The critical factors which impact the long term stability of device performance must be clarified more precisely. The wafer-level fabrication processes require further development to promote CNFET-based applications, such as gas sensors and superior transistors with ultralow power consumption.
